# Ultrasmall microdisk and microring lasers based on InAs/InGaAs/GaAs quantum dots

**DOI:** 10.1186/1556-276X-9-657

**Published:** 2014-12-04

**Authors:** Mikhail V Maximov, Natalia V Kryzhanovskaya, Alexey M Nadtochiy, Eduard I Moiseev, Ivan I Shostak, Andrey A Bogdanov, Zarina F Sadrieva, Alexey E Zhukov, Andrey A Lipovskii, Denis V Karpov, Janne Laukkanen, Juha Tommila

**Affiliations:** St. Petersburg Academic University, St. Petersburg, 194021 Russia; Ioffe Physical-Technical Institute, St. Petersburg, 194021 Russia; St. Petersburg State Polytechnical University, St. Petersburg, 195251 Russia; ITMO University, St. Petersburg, 197101 Russia; University of Eastern Finland, Joensuu, 80101 Finland; Optoelectronics Research Centre, Tampere University of Technology, Tampere, 33101 Finland

**Keywords:** Semiconductor quantum dots, Lasers, Microcavities, Microdisks, Microrings

## Abstract

**Abstract:**

Ultrasmall microring and microdisk lasers with an asymmetric air/GaAs/Al_0.98_Ga_0.02_As waveguide and an active region based on InAs/InGaAs/GaAs quantum dots emitting around 1.3 μm were fabricated and studied. The diameter *D* of the microrings and microdisks was either 2 or 1.5 μm, and the inner diameter *d* of the microrings varied from 20% to 70% of the outer diameter *D*. The microring with *D* = 2 μm and *d* = 0.8 μm demonstrated a threshold pump power as low as 1.8 μW at room temperature. Lasing was observed up to 100°C owing to the use of quantum dots providing high confinement energy both for electrons and holes. Tuning spectral positions of the whispering gallery modes via changing the inner diameters of the microrings was demonstrated.

**PACS:**

78.67.Hc; 42.55.Sa; 42.50.Pq; 78.55.Cr

**Electronic supplementary material:**

The online version of this article (doi:10.1186/1556-276X-9-657) contains supplementary material, which is available to authorized users.

## Background

Semiconductor microdisks (MDs) and microrings (MRs) are presently attracting increasing attention as key elements of future photonic integrated circuits. They can be used as low-threshold microlasers [1, 2], modulators [3], add/drop filters [4], etc. The targeted characteristics of such devices are high temperature stability, elevated operating temperatures, controllable spectral position of optical modes, and the possibility of current injection. In addition to that, a small size is very desirable to increase an intermode spectral interval, reduce an active region volume, and achieve high density of components in an optical circuit. However, fabrication of small-sized (of the order of charge carrier diffusion length) microcavities is challenging. This is because etching used for the fabrication, being performed through the active region, can result in non-radiative recombination of the carriers at the processed surfaces. This issue is especially crucial for the GaAs-based material system where the surface recombination rate is an order of magnitude higher than in the InP-based materials [5].

Strong surface recombination in GaAs-based material system can be eliminated via using self-organized quantum dots (QDs) as the active medium. Carrier lateral migration is inefficient even at 300 K, provided that the electron and hole confinement in QDs is sufficiently strong [6]. Thus, non-radiative recombination at sidewalls in such structures is weak, and etching can be done through the active region. Deeply etched narrow stripe QD lasers [7] and arrays of small-sized (0.2 μm) mesas [8] showed efficient emission at room temperature. To the best of our knowledge, the smallest QD microresonator, where room temperature lasing was demonstrated, had a diameter of about 2.1 μm [2]. In that structure, InAs/InGaAs QDs were used as the active region, and optical confinement was provided by air cladding in both sides (a suspended disk). Recently, we have demonstrated room temperature lasing with similar QDs in a 2.7-μm MR on an oxidized (AlGa)_*x*_O_*y*_ pedestal [9]. Both aforementioned designs provide strong optical confinement of the whispering gallery modes (WGMs) in vertical direction, but current injection is challenging.

In this paper, we present the results on elevated temperature lasing of QD MDs and MRs with diameters as small as 1.5 to 2 μm on a semiconductor pedestal.

## Methods

**Figure 1:**
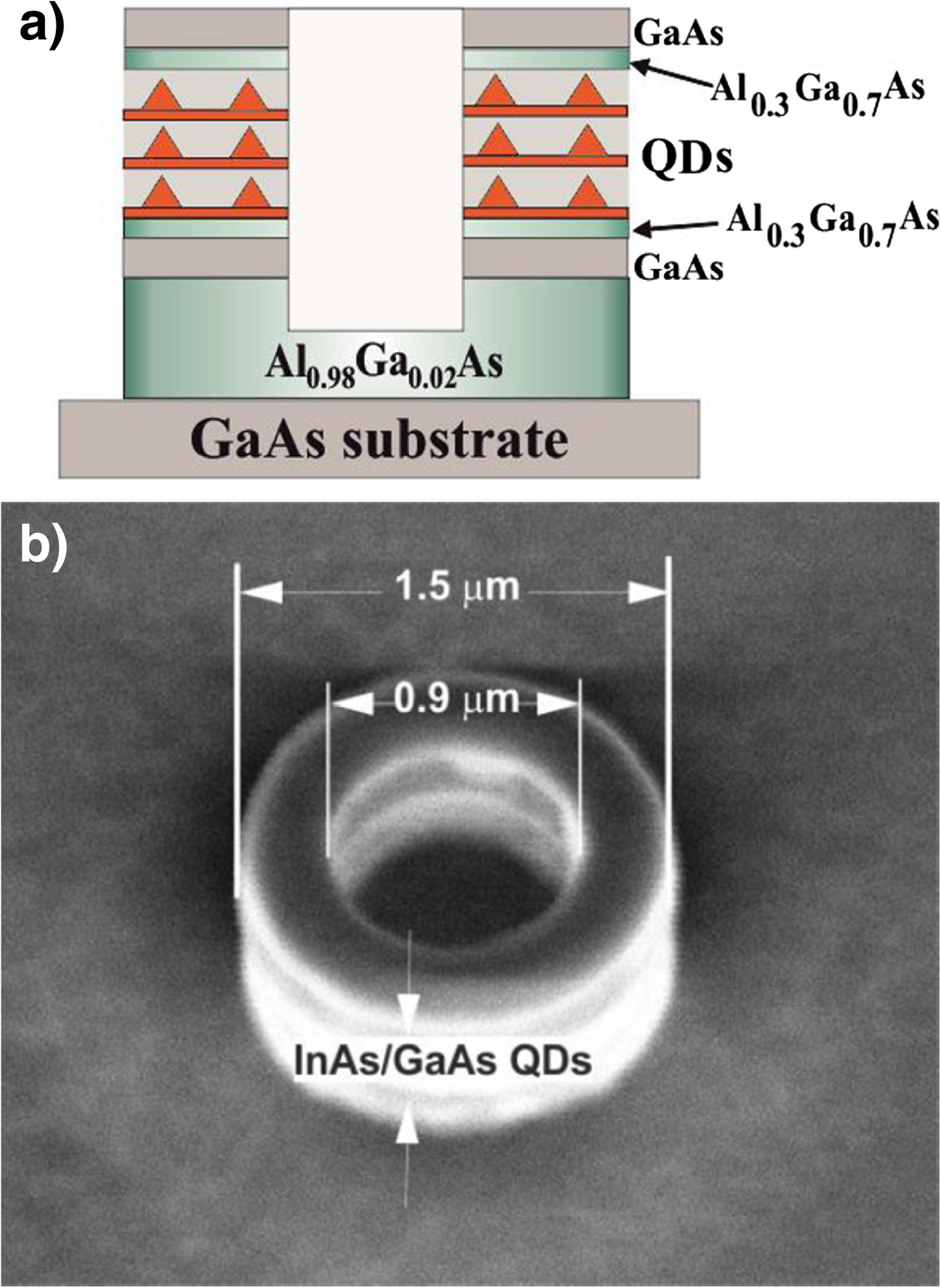
**Schematic layout (a) and scanning electron microscopy micrograph of a QD microring (b).**

An epitaxial wafer was grown using molecular beam epitaxy on a semi-insulating GaAs (100) substrate. The active region comprised five layers of InAs/In_0.15_Ga_0.85_As/GaAs QDs separated with 35-nm-thick GaAs spacers. The QDs were formed using directional migration of In atoms during overgrowth of the initial InAs Stranski-Krastanov QDs with an In_0.15_Ga_0.85_As capping layer [10]. Such QDs show high confinement energy of electrons and holes with respect to the capping layer and the GaAs matrix. The active region was placed in the middle of a GaAs layer confined from the both sides with 20-nm-thick Al_0.3_Ga_0.7_As barriers. The total thickness of the waveguide layer was about 350 nm. This waveguide layer was grown on top of the 450-nm-thick Al_0.98_Ga_0.02_As cladding layer that later formed the MD (or MR) pedestal (Figure 1a).

MDs and MRs with diameter (*D*) either 2 or 1.5 μm were fabricated using electron beam lithography (EBL) and reactive ion etching (Figure 1b). The inner diameter *d* of the microrings varied from 20% to 70% of the outer diameter. Fabrication of the MDs and MRs began with stacking two masks on the surface of the heterostructure. A main silicon dioxide (SiO_2_) mask was formed using atomic layer deposition, and a secondary mask of a negative resist (AZ® nLOF, MicroChemicals GmbH, Ulm, Germany) was deposited upon the SiO_2_ layer with spin coating. For EBL, we used a Gaussian beam vector scanning system which can provide the minimum spot size below 2.5 nm. The resist developing was done using pure AR 300–47 developer. After the development, the sample was rinsed in water for 30 s. The secondary mask was then employed to form the main mask. For this, we performed dry etching of SiO_2_ using reactive ion etching (RIE) system Plasmalab 80 (Oxford Plasma Technology, Yatton, Bristol). This mask was used in the GaAs etching process with RIE system Plasmalab 100 (Oxford Plasma Technology). No intentional oxidation of the Al_0.98_Ga_0.02_As pedestal was done. Such a design is compatible with current injection.

Microlasers were optically pumped with the second harmonic of CW-operating YAG:Nd laser (*λ* = 532 nm, 10 to 200 mW). An excitation power can be varied down to sub-microwatt level using absorptive neutral density optical filters. Optical emission was detected with a monochromator Horiba FHR 1000 and a CCD camera (Horiba Symphony, Edison, NJ, USA) (spectral resolution 30 pm). For temperature-dependent measurements, the structures were mounted onto a heated holder equipped with a temperature controller.

## Results and discussion

**Figure 2:**
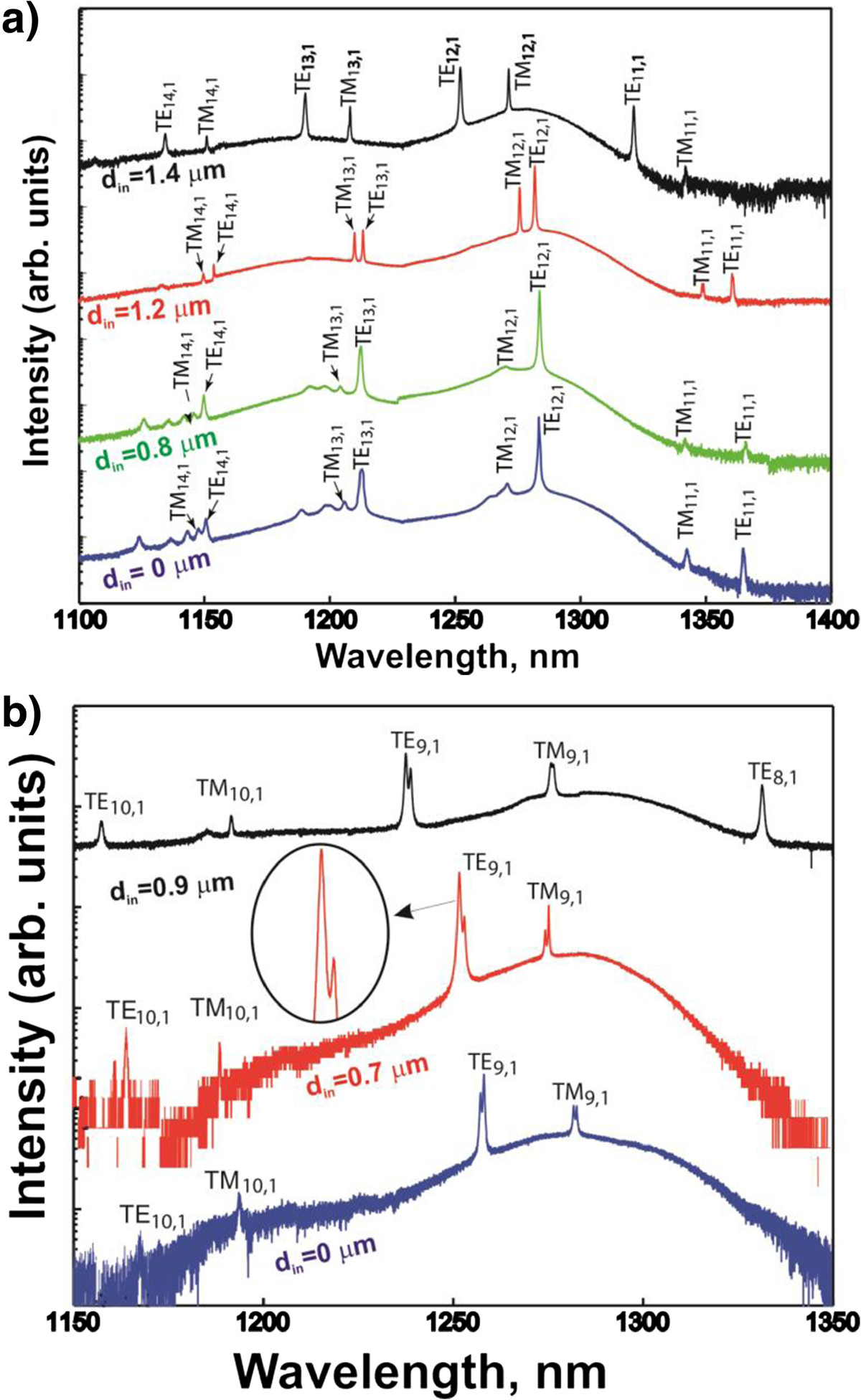
**Spectra of MDs and MRs with** ***D*** **= 2 μm (a) or** ***D*** **= 1.5 μm (b) and various** ***d*** **.**

Figure 2a, b compares the microphotoluminescence (μPL) spectra of the MDs and MRs with diameter *D* = 2 and 1.5 μm, respectively.

To identify the luminescence peaks observed, we numerically solved Maxwell’s equations by finite element method using COMSOL Multiphysics software. Owing to the axial symmetry, the problem simplifies from 3D to 2D case. Details of the simulation technique are described in [11]. In the simulation, three regions were considered: GaAs cavity, Al_0.98_Ga_0.02_As pedestal, and surrounding air (2 μm from both top and sidewall of the cavity). Radiation loss and imaginary parts of dielectric functions were not taken into consideration.

The observed peaks were identified on the base of our simulation as WGMs of different azimuthal orders as indicated in Figure 2. The observed splitting of the μPL lines, which is especially pronounced for the MDs and MRs with *D* = 1.5 μm, is due to surface roughness.

In the general case, the microcavities support both transverse electric (TE)-like and transverse magnetic (TM)-like modes [11–13]. It was shown [14, 15] that the TM-like modes *Q* factor has a step-like dependence on the cavity thickness and drastically decreases as the thickness is less than approximately 0.5*λ*/*n*[14], whereas the TE-like modes *Q* factor is still sufficiently high (here, *λ* is the emission wavelength and *n* is the refractive index). Since in our case the thickness is about 0.9*λ*/*n*, the microresonators under study can support not only TE-like but also TM-like modes.

**Figure 3:**
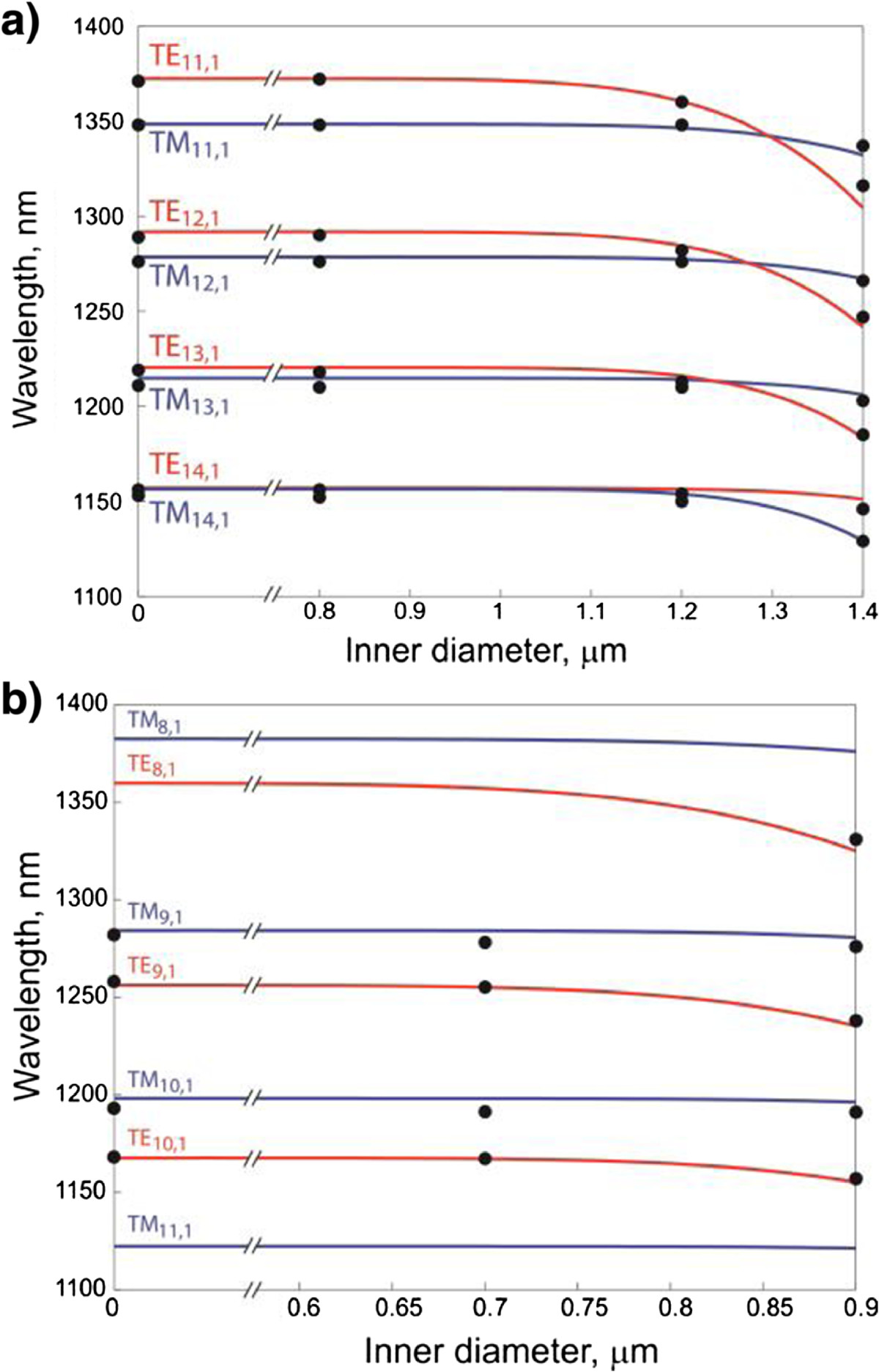
**Dependence of mode wavelengths for** ***D*** **= 2 μm (a) and** ***D*** **= 1.5 μm (b) on inner diameter.**

The positions of the most intensive peaks in the μPL spectra are best fitted to the wavelengths of TE-like and TM-like modes of the first radial order (Figures 2 and 3). Some disagreement between the experimental (curves) and theoretical (symbols) results can be attributed to the sample overheating under CW excitation, uncertainty in optical constants of QDs and surrounding layers, and to the fact that MD and MR geometrical parameters (layer thicknesses, diameters, shape) are estimated from SEM images within ±5% precision. Weaker peaks in the spectra of the MD with *D* = 2 μm and MR with *D* = 2 μm and *d* = 0.8 μm (not marked in Figure 2a) are attributed to the modes of higher radial order. This identification is confirmed by the fact that they are nearly disappeared in the MR with larger hole diameter *d* = 1.2 μm and are not observed in the MR with *d* = 1.4 μm.

**Figure 4:**
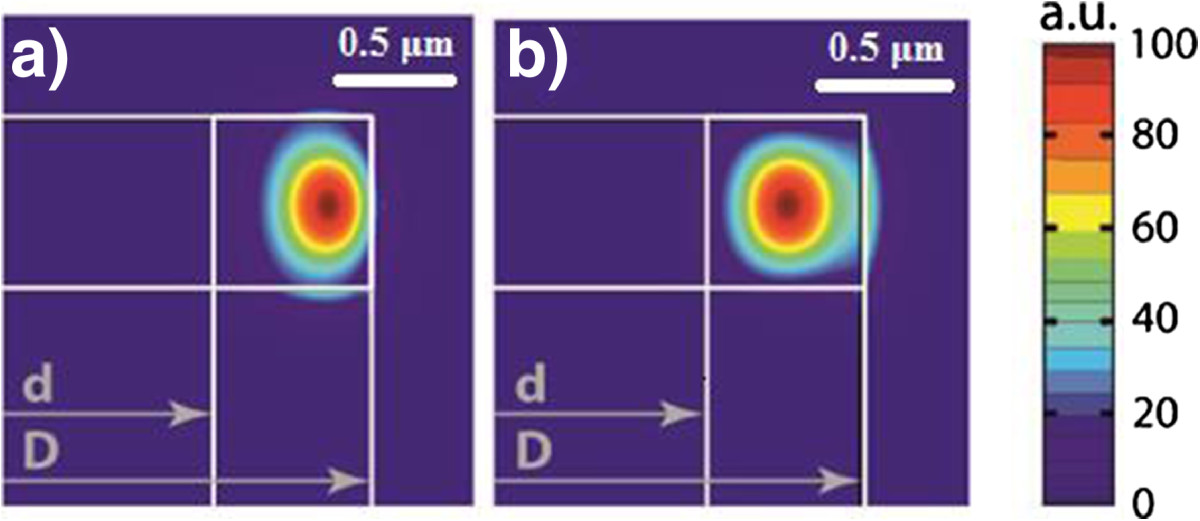
**Spatial distribution of electric field magnitude of TM**_**10,1**_**(a) and TE**_**10,1**_**(b) modes (** ***D*** **= 1.5 μm,** ***d*** **= 0.9 μm).**

If the inner diameter of the MRs is small, the wavelengths of WGMs only weakly change with respect to those of the MDs (Figure 3a, b). The holes of small diameters have negligible effect because WGMs of the first radial order mainly concentrate at the periphery of MRs (at distances larger than 0.4 radius from the center). With further increasing in the inner diameter, the WGM wavelengths decrease. The observed wavelength shift is less for modes of higher azimuthal orders (*m*), because the larger *m* the stronger the mode localization in the vicinity of the outer boundary. TM-like modes are localized closer to the outer boundary of MRs as compared to TE-like modes (Figure 4), and therefore, their wavelength shift is less pronounced.

**Figure 5:**
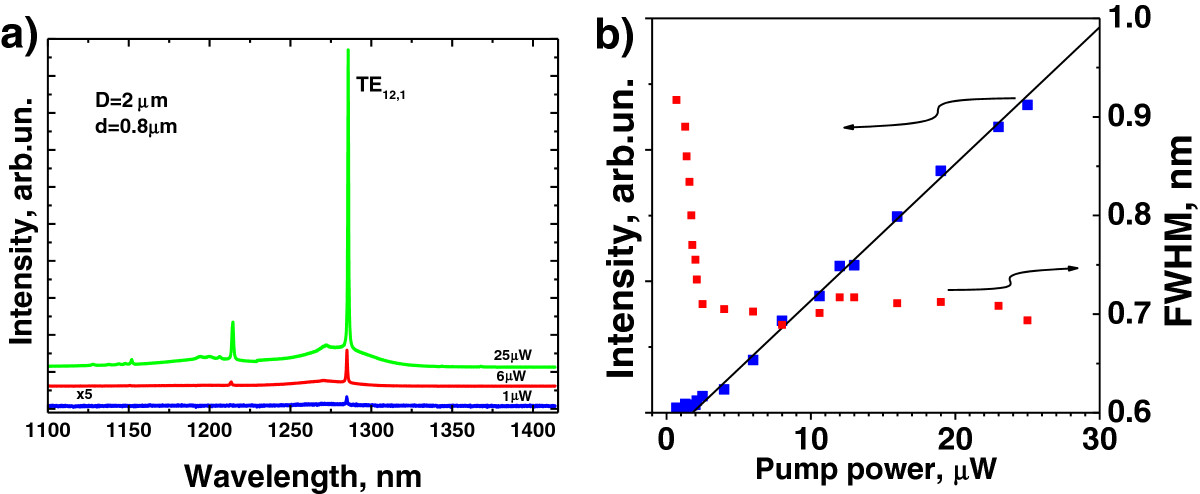
**Microphotoluminescence spectra (a) and dependence of TE**_**12,1**_ **mode intensity and linewidth (b) on the excitation power.**

Figure 5a shows the microphotoluminescence spectra for the MR with *D* = 2 μm and *d* = 0.8 μm at various pump powers. The most intensive line corresponds to WGM TE_12,1_ whose wavelength approximately coincides with the spectral position of the gain maximum. Figure 5b shows the dependence of the TE_12,1_ mode intensity on the excitation power (‘light-in-light-out’ characteristic). To determine the mode intensity, we subtracted the background due to broad spontaneous emission and then integrated the spectrum of the mode TE_12,1_. The light-in-light-out curve shows a pronounced threshold kink at 1.8 μW, and approximately at the same value of pump power, a significant linewidth narrowing takes place. Both effects evidence laser action for the mode TE_12,1_. The measured value of threshold pump power is comparable with the best results obtained in QD microlasers. An incident pump power threshold of approximately 2.5 μW (about 0.4 μW of the absorbed power) and an absorbed power threshold of 1.1 μW have been reported, respectively, for a photonic crystal nanocavity laser [15] and an air-cladded microdisk of 2.1 μm in diameter [2].

**Figure 6:**
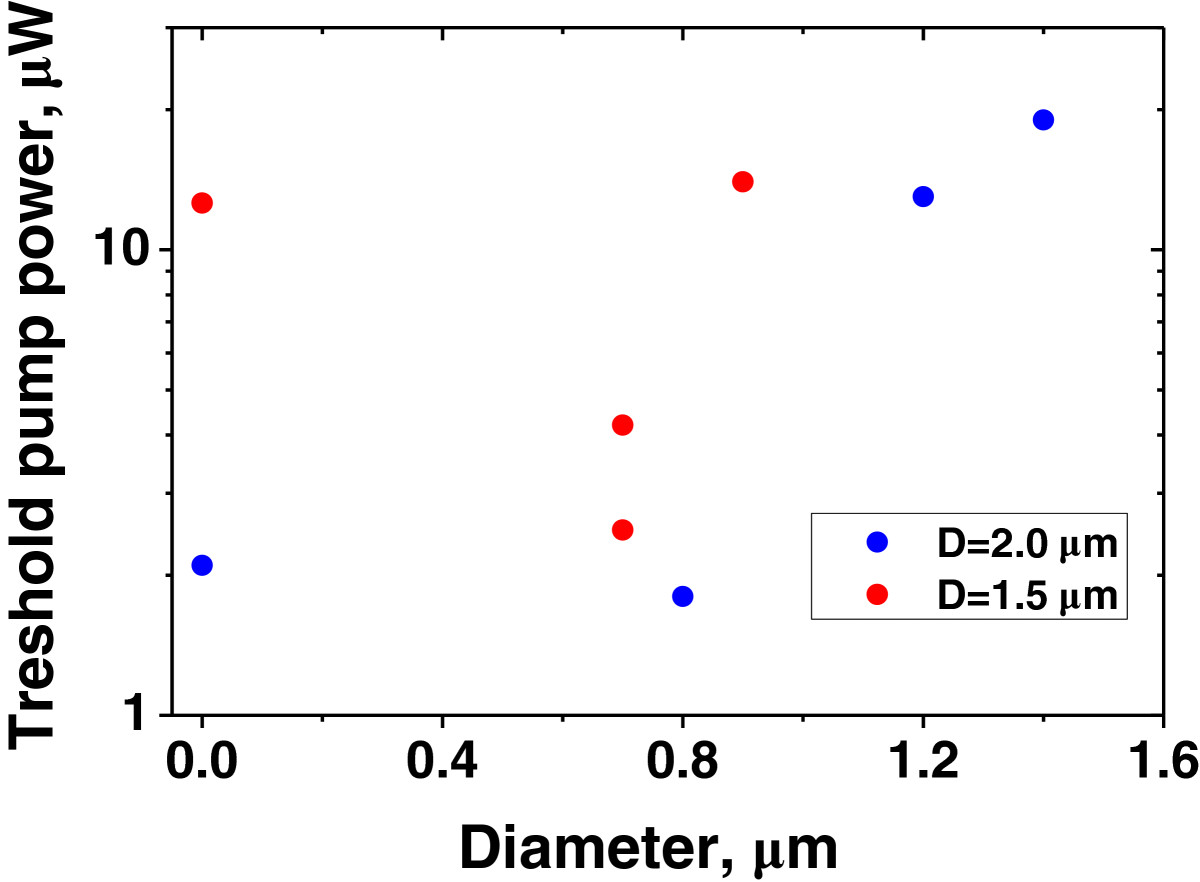
**The threshold pump power against inner MR diameter.**

The evolution of threshold pump powers for the MRs with *D* = 2 and 1.5 μm with the increase in inner MR diameters is summarized in Figure 6. The threshold powers were determined using the dependencies of the intensities and linewidths of the dominant modes on excitation power (similar to Figure 5b). The lasing threshold slightly decreases as the inner MR diameter *d* increases from zero (MD) to 0.7-0.8 μm most likely due the decrease in the active region volume. However further increase in *d* results in a rapid growth of the generation threshold, which can be explained by increased non-radiative recombination at sidewalls (waveguide width decreases to 0.4 μm) as well as by enhanced light scattering at microring wall roughness.

For the 1.5 μm in diameter microcavities, the lowest threshold power of about 2.5 μW was measured in the MRs with *d* = 0.7 μm. Higher threshold as compared to the MRs with *D* = 2 μm is probably due to higher scattering losses and increased contribution of non-radiative recombination at sidewalls.

The lasing threshold of a certain WGMs depends on a detuning between the wavelength of this mode and the maximum of gain spectrum. The wavelength of the gain maximum approximately follows the temperature dependence of the GaAs bandgap (approximately 0.5 nm/K), while the temperature shift of the WGM resonant wavelength is primarily governed by the temperature variation of the refractive index and is slower (<0.1 nm/K). Thus, the detuning is changing with temperature and may result in a non-monotonic temperature dependence of the threshold power for WGMs.

**Figure 7:**
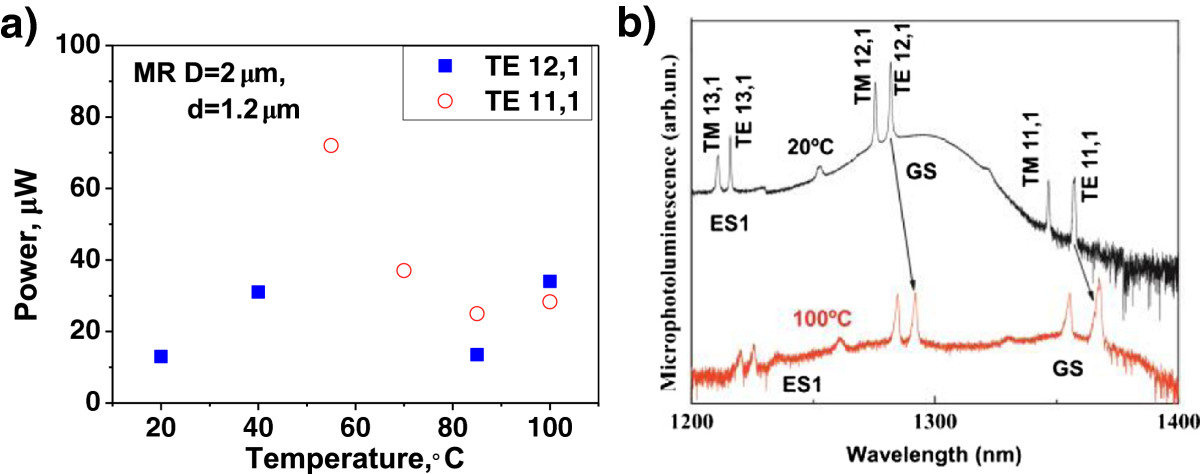
**Temperature variation of the threshold power (a) and microphotoluminescence spectra (b).**

Figure 7a summarizes the temperature variation of the threshold power of the two lowest threshold modes (TE_12,1_ and TE_11,1_) for the MRs with *D* = 2 μm and *d* = 1.2 μm. At room temperature, the wavelength of TE_12,1_ mode approximately corresponds to the spectral position of the ground state gain maximum (Figure 7b), and the threshold power is low. With temperature increase, a red shift of the gain peak occurs. Correspondingly, the position of TE_12,1_ mode moves towards the short-wave side of the gain curve, and the threshold power increases. In the 45°C to 85°C interval, the wavelength of this mode is in between the peaks of the ground and excited states, and the lasing becomes unattainable. At 50°C, the wavelength of TE_11,1_ WGMs falls on the long-wave tail of the ground state transition, and lasing via this mode starts with a high threshold power. With temperature increase, the position of TE_11,1_ mode moves towards the maximum of the ground state gain, and the threshold power drops. At 85°C and 100°C, the wavelengths of TE_12,1_ and TE_11,1_ modes are within the range of QD excited state and ground state transitions, correspondingly. The lasing simultaneously occurs via both modes with comparable threshold power levels.

## Conclusions

Microrings and microdisks with a semiconductor AlGaAs pedestal were fabricated. The active region of the lasers was based on InAs/InGaAs/GaAs quantum dots emitting near 1.3 μm with high confinement energy for electrons and holes. Whispering gallery modes of the microrings and microdisks were numerically simulated. The positions of the most intensive peaks were shown to fit best to the energies of TE-like and TM-like modes of the first radial order. The WGM resonant wavelengths decrease with increasing inner hole diameter *d*; the tuning range can be up to 50 nm. The threshold power depends on the MR inner diameter and reaches its minimum of 1.8 μW at *d* = 0.8 μm. Lasing up to 100°C is achieved. The temperature dependence of the threshold power for a certain WGMs is determined not only by the temperature dependence of the gain spectrum but also by the position of this mode at the gain curve (detuning).

## Authors’ information

MM holds a leading researcher position at St. Petersburg Academic University, a leading researcher position at Ioffe Physical-Technical Institute, and a leading researcher position at St. Petersburg State Polytechnical University. NK holds a senior researcher position at St. Petersburg Academic University and a senior researcher position at St. Petersburg State Polytechnical University. AN holds a senior researcher position at St. Petersburg Academic University and a senior researcher position at St. Petersburg State Polytechnical University. EM is a Master student at St. Petersburg Academic University. IS is a Master student at St. Petersburg Academic University. AB holds a senior researcher position at St. Petersburg Academic University, a senior researcher position at St. Petersburg State Polytechnical University, and a senior researcher position at ITMO University. ZS is a Master student at St. Petersburg Academic University. AZ holds a provost position at St. Petersburg Academic University and a professor position at St. Petersburg State Polytechnical University. AL holds a professor position at St. Petersburg Academic University and a professor position at St. Petersburg State Polytechnical University. DK holds a PhD degree at the University of Eastern Finland. JL holds a senior researcher position at the University of Eastern Finland. JT holds a senior researcher position at Tampere University of Technology.

## Authors’ original submitted files for images

Below are the links to the authors’ original submitted files for images. Authors’ original file for figure 1 Authors’ original file for figure 2 Authors’ original file for figure 3 Authors’ original file for figure 4 Authors’ original file for figure 5 Authors’ original file for figure 6 Authors’ original file for figure 7 Authors’ original file for figure 8 Authors’ original file for figure 9 Authors’ original file for figure 10 Authors’ original file for figure 11 Authors’ original file for figure 12

## Abbreviations

EBL: electron beam lithography
MDs: microdisks
MRs: microrings
μPL: micro-photoluminescence
QDs: quantum dots
WGMs: whispering gallery modes.
