# Supplementary figures and images for: Ultrasmall microdisk and microring lasers based on InAs/InGaAs/GaAs quantum dots
Source: Nanoscale Res Lett. 2014 Dec 4;9:657. doi: 10.1186/1556-276X-9-657 (PMC4883690; doi:10.1186/1556-276X-9-657)

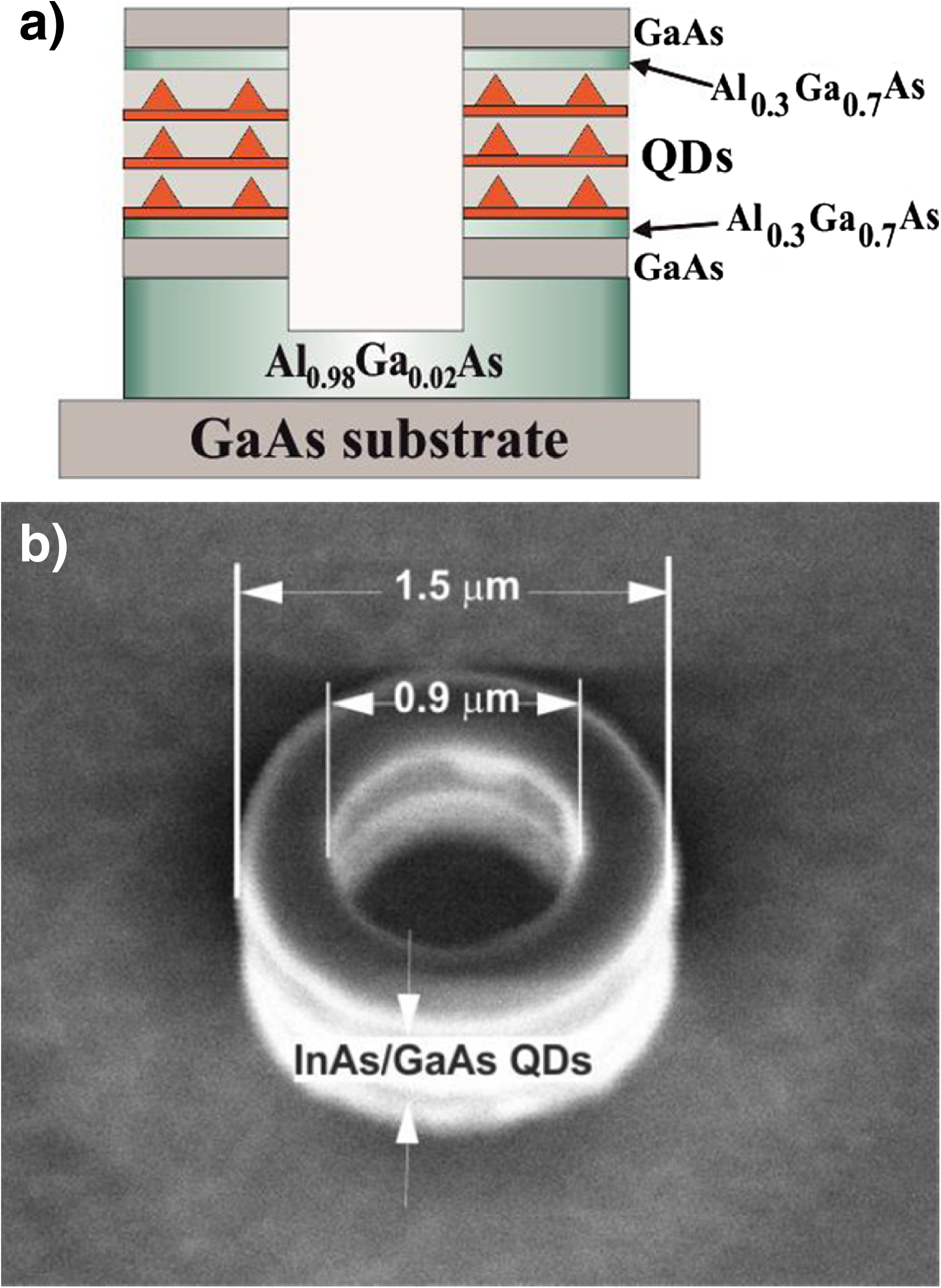

Supplement: Supplementary file 1 — Authors’ original file for figure 1 [file 11671_2014_3266_MOESM1_ESM.tiff]

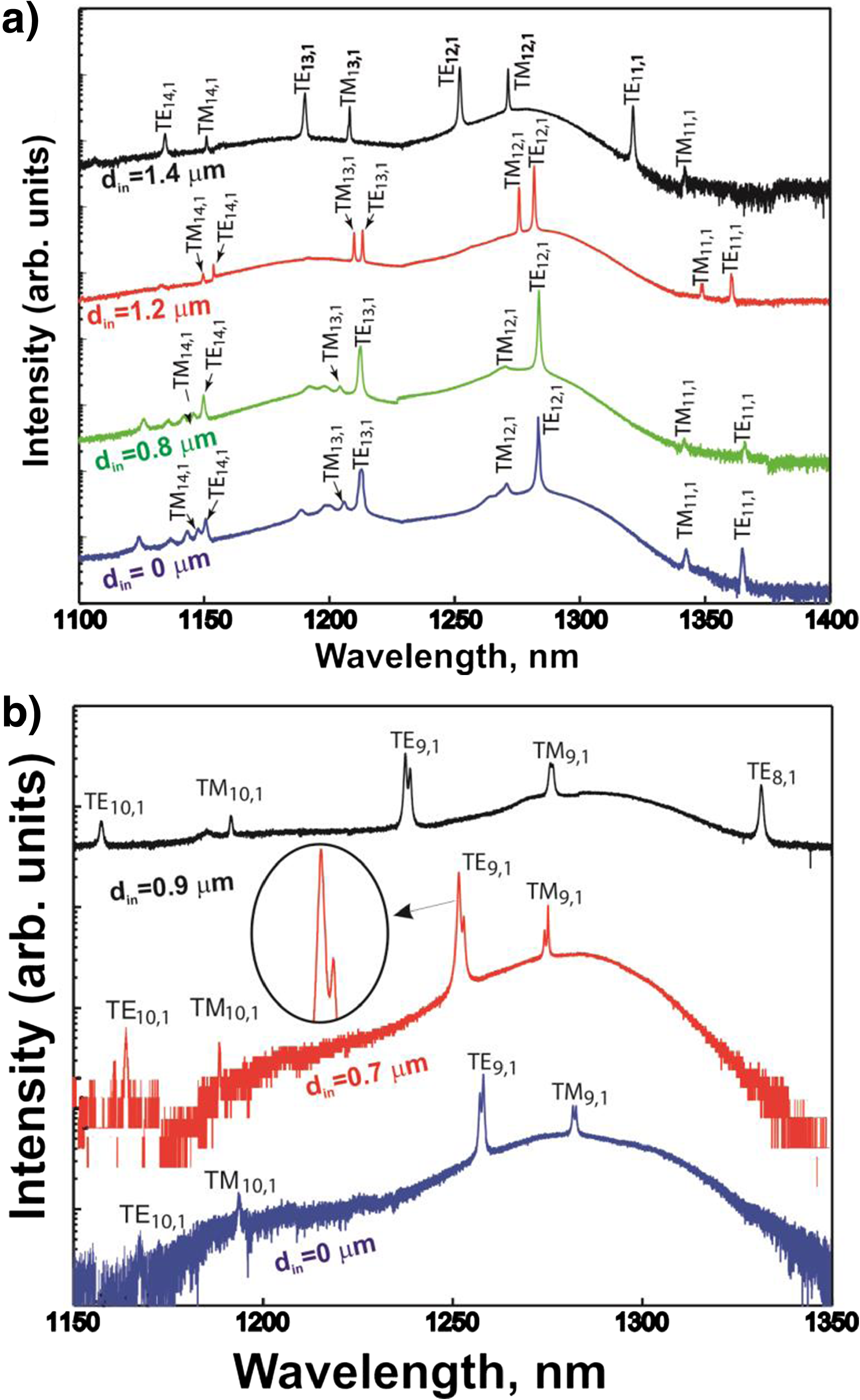

Supplement: Supplementary file 2 — Authors’ original file for figure 2 [file 11671_2014_3266_MOESM2_ESM.tiff]

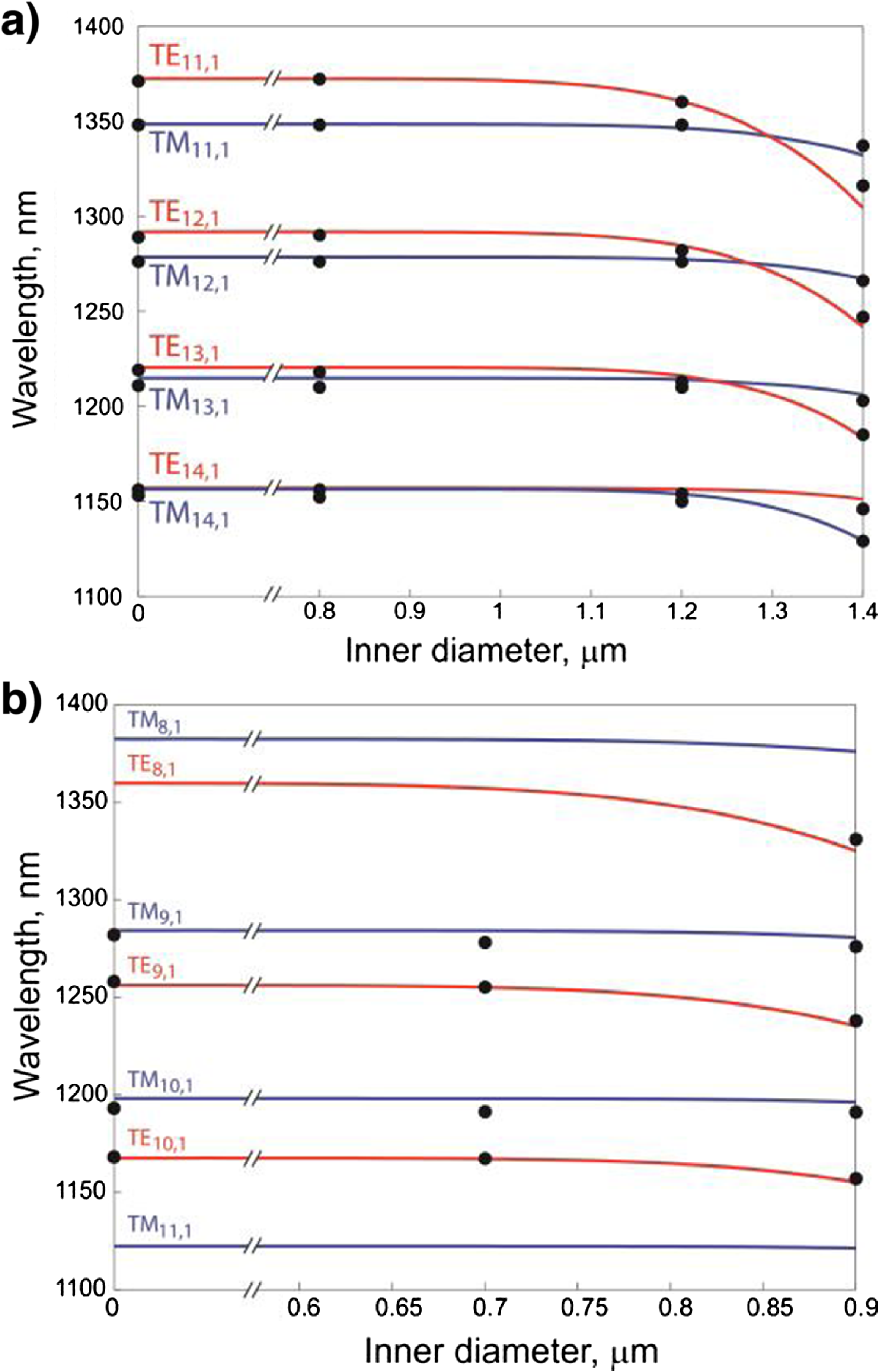

Supplement: Supplementary file 3 — Authors’ original file for figure 3 [file 11671_2014_3266_MOESM3_ESM.tiff]

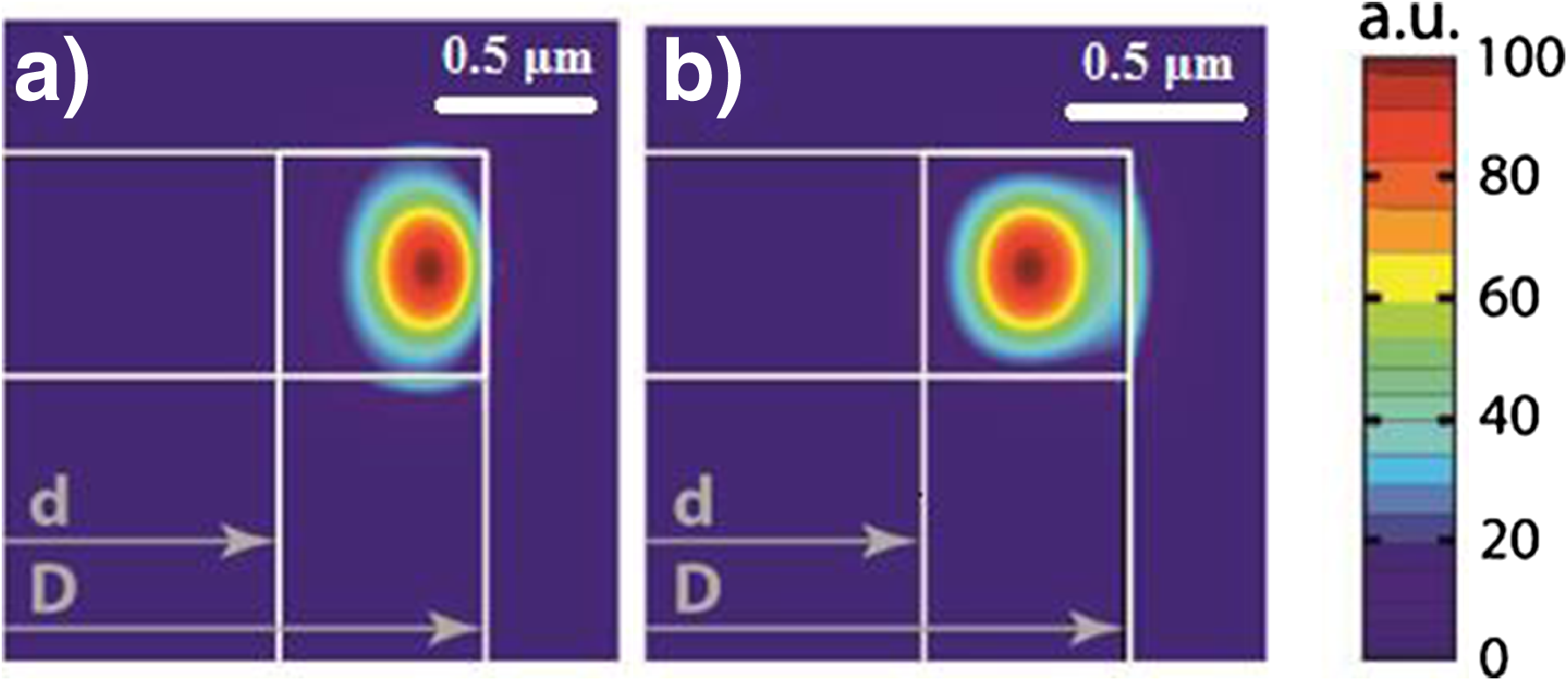

Supplement: Supplementary file 4 — Authors’ original file for figure 4 [file 11671_2014_3266_MOESM4_ESM.tiff]

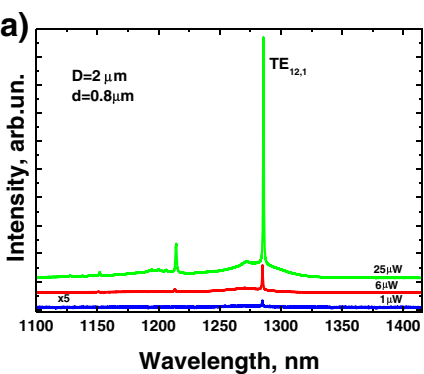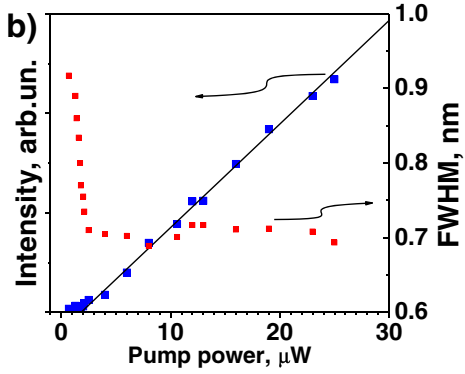

Supplement: Supplementary file 5 — Authors’ original file for figure 5 [file 11671_2014_3266_MOESM5_ESM.pdf]

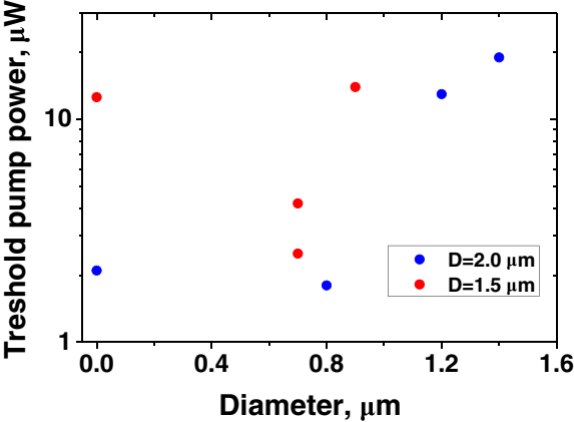

Supplement: Supplementary file 6 — Authors’ original file for figure 6 [file 11671_2014_3266_MOESM6_ESM.pdf]

a)

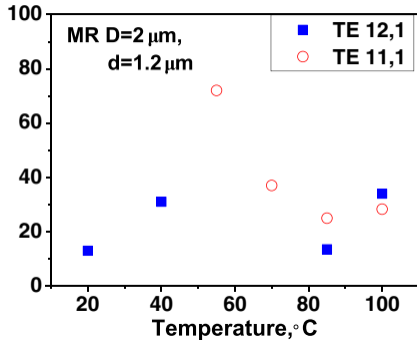

b)

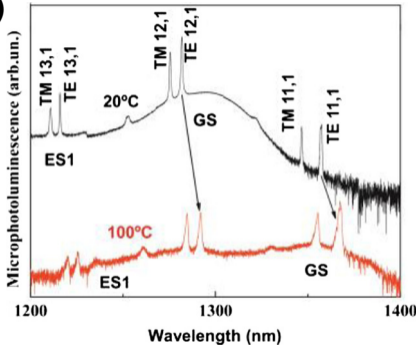

Supplement: Supplementary file 7 — Authors’ original file for figure 7 [file 11671_2014_3266_MOESM7_ESM.pdf]

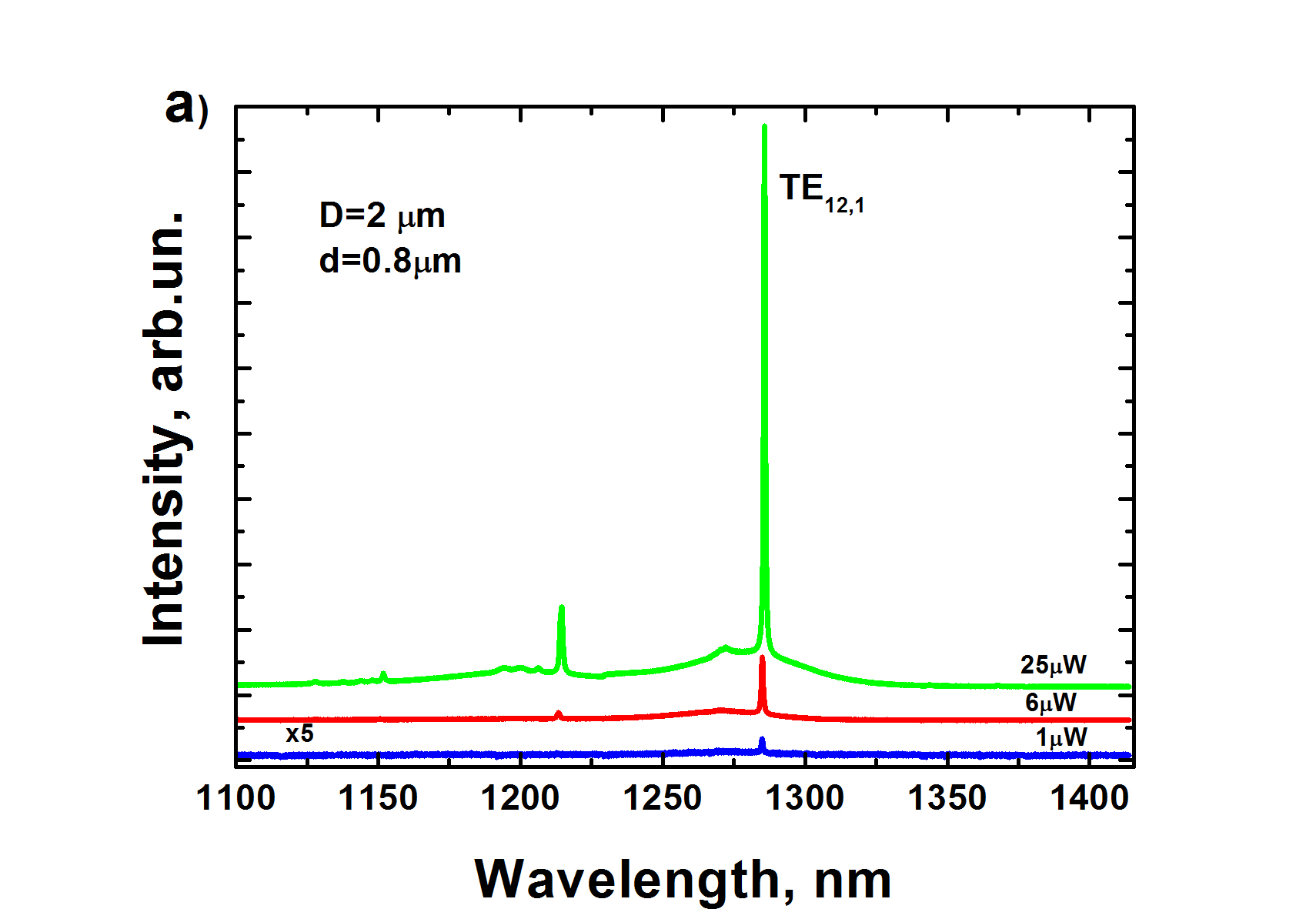

Supplement: Supplementary file 8 — Authors’ original file for figure 8 [file 11671_2014_3266_MOESM8_ESM.jpeg]

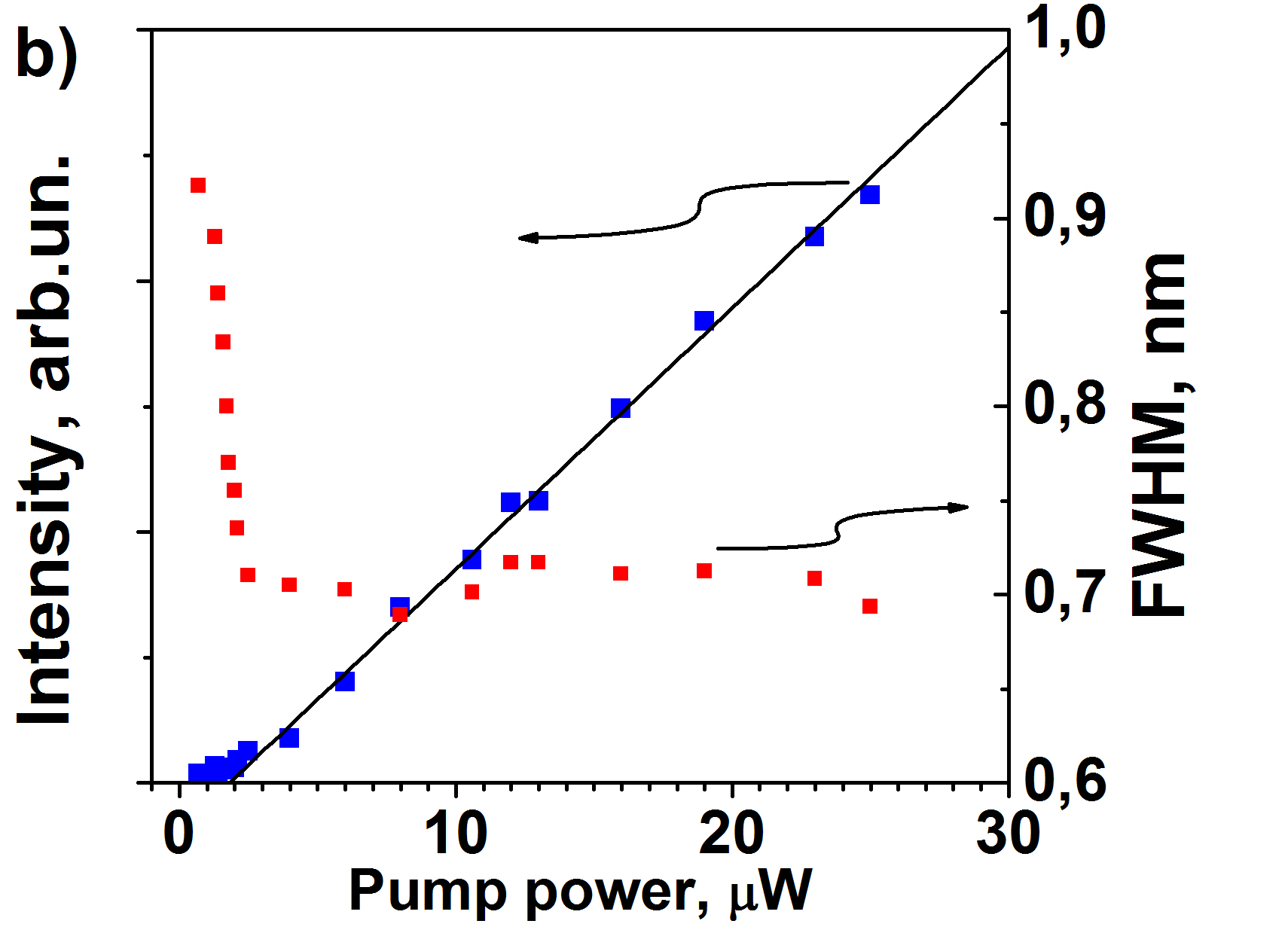

Supplement: Supplementary file 9 — Authors’ original file for figure 9 [file 11671_2014_3266_MOESM9_ESM.jpeg]

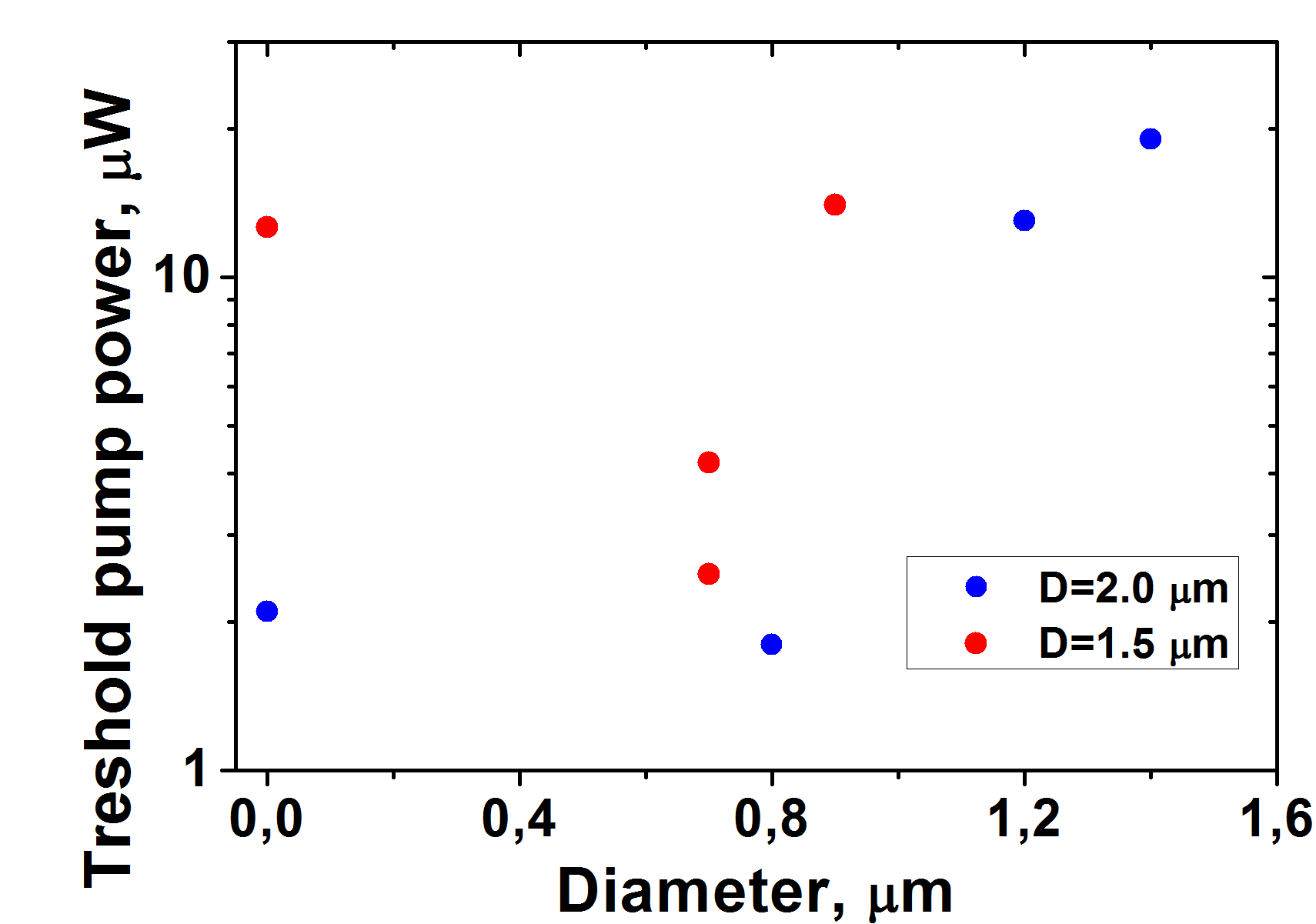

Supplement: Supplementary file 10 — Authors’ original file for figure 10 [file 11671_2014_3266_MOESM10_ESM.jpeg]

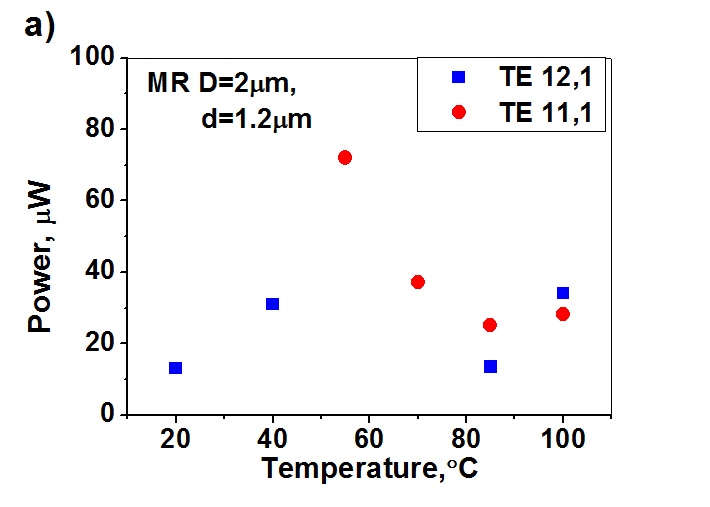

Supplement: Supplementary file 11 — Authors’ original file for figure 11 [file 11671_2014_3266_MOESM11_ESM.jpeg]

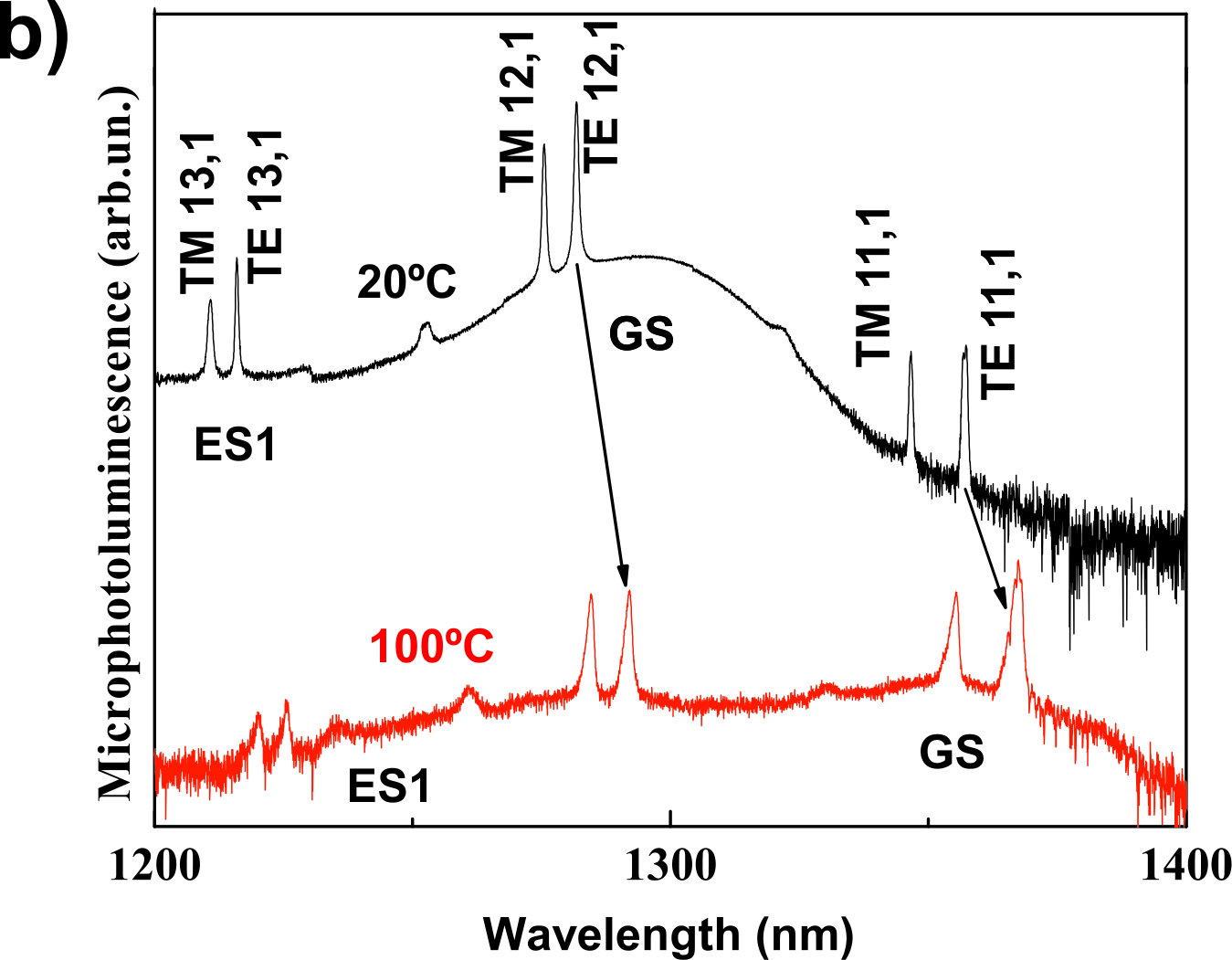

Supplement: Supplementary file 12 — Authors’ original file for figure 12 [file 11671_2014_3266_MOESM12_ESM.jpeg]
